# 

**Published:** 1891-04

**Authors:** 


**Treatment of Incontinence of Urine in Females.**—*The College and Clinical Record* reproduces an article from the *International Journal of Surgery*, January, 1891, by H. Marion Sims, M. D., of New York, in which he describes a new treatment for the most disagreeable and annoying condition of the bladder known as Contraction of the Viscus, which is due to the hypertrophy of its muscular coat. This condition is brought about by neglect in infancy and childhood. The bladder is never permitted to hold its urine for any length of time. Children give the history of wetting the bed, and not holding the urine long during the daytime. This habit of wetting the bed followed the patient through childhood to maturity, when it is often found that the bladder will not hold more than one ounce of water. The sphincter muscle of the bladder is in a paralyzed condition. No inflammation exists.

The treatment consists in dilatation with warm water. A silver catheter, to which a rubber tube is attached, and to this a Davidson syringe, make up the instruments needed in this operation.

The dilatation is repeated daily and continued for several months until the bladder will hold eighteen or twenty ounces of fluid. This report is based on sixteen cases all treated successfully.

